# Remarks by Dr. Reynell Coates

**Published:** 1842-07-30

**Authors:** 


[*Remarks.*—The foregoing paper may prove useful by impressing the importance of a measure which, though by no means novel, has been but seldom practised. We allude to the provision of proper support to the femur in fractures of the neck. It is now no longer doubtful that osseous or very firm chondroid union has occasionally occurred in fractures within the capsular ligament, and the possibility of such a result should render us extremely careful to favour accuracy of coaptation by all possible means. We believe that on a well adjusted bed, with the limb in the straight position, the weight of the bone can have little influence in carrying it away from the superior fragment, that weight being far more than compensated by the tonicity of the greater psoas and the iliacus muscles: but the action of these muscles may prove insufficient to counteract that of the three glutii, and especially the great gluteus, which tend to draw the trochanter major, and, with it, the shaft of the femur, backwards. It is, therefore, desirable, in fractures of the neck of the thigh bone, that the upper part of the thigh should be properly supported by means of a broad compress duly graduated—a measure that need not interfere in any degree with the permanence of the necessary extension and counter-extension, unless the limb itself be incumbered with useless bandages, as is usually directed in most of the systematic treatises.

To the flexed position, as recommended by the writer of the article under notice, there exists a most serious objection. By the relaxation of the psoas and iliacus, and the partial extension of the glutii muscles, we at once diminish very seriously the only natural force which tends to prevent the retreat of the superior extremity of the inferior fragment, while we materially increase the already too powerful muscular action tending to produce this deformity. To this difficulty is superadded the awkwardness, if not impossibility of efficiently counteracting the disposition to a retrocession by means of a compress. The horse hair pad, recommended in the paper, is infinitely less certain in its action than is a properly graduated compress based upon an ordinary mattrass, and acting upon the thigh alone in the extended position.

The employment of a band around the pelvis and trochanter major appears to us totally unnecessary when the limb is treated in the extended position; for, there exists no force tending to separate the fragments by carrying the base of the neck *outwards* from the superior fragment, and the tonicity of the adductors of the thigh, including the glutii, is quite sufficient, unless in cases of paralysis, to preserve the coaptation of the fragments when permanently extended to the proper degree, duly supported, and prevented from forming an angular deformity by placing the foot in a proper position. The band recommended is liable to the objection of concealing the condition of the parts, and proving uselessly annoying to the patient; for, when the extended position is selected, it answers no legitimate indication in the case.

Though the flexed position is never likely to find many advocates in America, the practice of Pott being here generally discarded, it may be well to remark that, as the head of the bone in these cases does not necessarily follow the rotation of the base of the neck in the movement of flexing the thigh upon the pelvis, there is always danger that, in case of union being effected in that position, *the power of extending the thigh* after the cure may be permanently limited. To this we may add that the due extension and counter-extension of the limb—which constitute by far the most important means of coaptation, though they are scarcely touched upon by the writer under review—cannot be effected with any precision in the flexed or double flexed position.

In one of the cases mentioned by the writer, a persistent immobility of the knee joint is attributed to the straight position, and the bandages applied to the limb. We believe that no excusable reason can be given for the application of any bandages directly to the limb in any uncomplicated fractures of the thigh, except in those which, being oblique from before backwards, are seated within two or three inches above the origin of the gastrocnemii muscles. As for the stiffness resulting from the long continued rest of the knee and ankle joints, it is nearly as likely to occur in the one attitude—properly preserved—as in the other; its nature, temporary character and cure, whenever it happens to occur, are perfectly understood; and the experience of France and the United States, as well as that of some modern English surgeons, proves that it is not a valid objection to the straight position. It is, indeed, somewhat curious that ancient custom should so far influence the judgment of a writer who reports two beautifully successful cures of an accident once universally, and still generally deemed irremediable; that he should advocate double flexion of the limb, when, in both his cases, *the complete extension of the leg and thigh was the method actually practised.* We feel perfectly assured, upon broad mechanical principles, that if the directions given at the conclusion of the paper had been followed, the coaptation would have been much less perfect, and the results consequently less happy.

That the supply of blood to the superior fragment in fractures within the capsule is extremely limited, is sufficiently proved by the atrophy of the round ligament and the fragment itself, the absorption of the stump of the neck attached to the head of the femur, the deficiency or sparcity of ossific deposit thereon, and other well known phenomena, usually observed *post mortem* in old cases of this character. The cure, then, must mainly depend upon the rapidity with which the circulation in the superior fragment can be restored; and the only source from which new vascular connections to a sufficient extent can be rapidly derived, is from the capillaries of the cancellated structure of the base of the neck. From these must the mass of the first bond of union be supplied and organised; for, the doctrine of certain hypertheorists who neglect the study of the vicarious actions of our various organs—a doctrine which teaches that most of these vessels are incapable of secreting bone, because, in health, they are mainly engaged in the secretion of the medulla—will be immediately repudiated by any uncommitted examiner who studies the early history of reunion in fractures. In order that proper anastomoses may be established by the almost unaided energies of one fragment, it becomes all important that the coaptation should be rendered as accurate and permanent as possible. Disturbing causes, of very little importance in most fractures, may, therefore, prove fatal to success in those occurring within the capsule; and the paper under notice is rendered highly valuable, by calling attention to one very important indication in effecting coaptation; but we cannot agree with the writer in thinking that the intervention of the capsular ligament between the fragments is so light an evil as he represents it. The intervention of any substance other than free cellular tissue, between the fragments in an ordinary fracture, always greatly enhances the labour of cure; and when *ligamentous matter* is so placed, the danger of pseudarthrosis is imminent. In fractures within the capsule, we must regard such an occurrence, in addition to the other extreme difficulties of the case, as necessarily precluding all reasonable hope of firm union in fractures within the capsule: fortunately, the chances of such a disaster are few—much fewer, we think, than some authors suppose; and when due allowance is made for the former neglect of accurate coaptation, the still existing prejudices of certain schools, in relation to the attitude of the limb, and the general impression of the hopelessness of these accidents, which often leads to carelessness in the treatment, we cannot avoid the hope and confident anticipation that the cure, without lameness, of fractures of the neck of the femur within the capsule, will soon cease to be regarded as an astonishing result.] R. C.

